# Estimation of the *RNU2* macrosatellite mutation rate by *BRCA1* mutation tracing

**DOI:** 10.1093/nar/gku639

**Published:** 2014-07-17

**Authors:** Chloé Tessereau, Yann Lesecque, Nastasia Monnet, Monique Buisson, Laure Barjhoux, Mélanie Léoné, Bingjian Feng, David E. Goldgar, Olga M. Sinilnikova, Sylvain Mousset, Laurent Duret, Sylvie Mazoyer

**Affiliations:** 1 Genetics of Breast Cancer Team, Cancer Research Centre of Lyon, CNRS UMR5286, Inserm U1052, Université Lyon 1, Centre Léon Bérard, Lyon, France; 2 Genomic Vision, Bagneux, Paris, France; 3 Laboratoire de Biométrie et Biologie Evolutive, CNRS UMR5558, Université Lyon 1, France; 4 Unité Mixte de Génétique Constitutionnelle des Cancers Fréquents, Hospices Civils de Lyon/Centre Léon Bérard, Lyon, France; 5 Department of Dermatology and Huntsman Cancer Institute University of Utah School of Medicine, Salt Lake City, Utah, USA

## Abstract

Large tandem repeat sequences have been poorly investigated as severe technical limitations and their frequent absence from the genome reference hinder their analysis. Extensive allelotyping of this class of variation has not been possible until now and their mutational dynamics are still poorly known. In order to estimate the mutation rate of a macrosatellite, we analysed in detail the *RNU2* locus, which displays at least 50 different alleles containing 5-82 copies of a 6.1 kb repeat unit. Mining data from the 1000 Genomes Project allowed us to precisely estimate copy numbers of the *RNU2* repeat unit using read depth of coverage. This further revealed significantly different mean values in various recent modern human populations, favoring a scenario of fast evolution of this locus. Its proximity to a disease gene with numerous founder mutations, *BRCA1*, within the same linkage disequilibrium block, offered the unique opportunity to trace *RNU2* arrays over a large timescale. Analysis of the transmission of *RNU2* arrays associated with one ‘private’ mutation in an extended kindred and four founder mutations in multiple kindreds gave an estimation by maximum likelihood of 5 × 10^−3^ mutations per generation, which is close to that of microsatellites.

## INTRODUCTION

Tandem repeat DNA sequences, also called satellite DNA, represent a high proportion of the human genome. Due to their unique structural features, they are likely to contribute to genetic diversity and thereby to the variability of human traits, including diseases. The most extensively studied tandem repeat DNA sequences so far have been microsatellites (composed of units from 1 to 10 bp), in particular those involved in trinucleotide repeat disorders, and minisatellites (units from 10 to a few hundreds bp long). Comparatively, little is known about tandemly repeated sequences longer than 1 kb, also known as multi-allelic tandem copy number variants (CNVs) or macrosatellites. Although macrosatellites constitute a sizeable fraction of large CNVs (1), can encompass large genomic intervals and are highly enriched with gene content, their impact on human genome plasticity has been poorly investigated despite the association of some arrays with disease susceptibility (2–4). This can be explained by the fact that they are very difficult to genotype directly using genome-wide platforms and are poorly tagged by single-nucleotide polymorphisms (SNPs) (5–7). Moreover, such regions are difficult to sequence and assemble and, as a result, they tend to be omitted from the human reference assembly (1), which excludes them from genome-wide investigations.

Nowadays, less than a dozen macrosatellites have been characterized although several attempts to identify new tandem repeats in the sequenced human genome have been made (1,8–9). The level of polymorphism of most of them has been evaluated on a low number of individuals using relatively low-resolution techniques such as pulse field- or field inversion-gel electrophoresis (PFGE or FIGE), yet these were sufficient to reveal one of the highest degrees of allelic diversity in the human genome, attesting their rapid evolution. Likewise, the mutational rate of macrosatellites has been studied only in a limited number of meioses. A high meiotic mutation rate (8.3 × 10^−2^ mutations per generation) was found for two macrosatellites, DXZ4 and RS447 (9,10), when analysing 24 and 60 parents-to-offspring transmissions, respectively, but subsequently no intergenerational RS447 copy number alteration could be observed in 120 meioses (11).

The *RNU2* macrosatellite has been found to be hypervariable, with a 6.1 kb-long unit repeated from 5 to 82 times in a total of ∼ 300 individuals of Caucasian, Asian and African origin (11,12). In a previous study that we conducted on 41 Caucasian individuals, the heterozygosity level of this microsatellite reached 98%. The *RNU2* locus has been localized by FISH at 17q21, close to the breast cancer predisposing gene *BRCA1* (13,14), precisely at a distance of 124 kb (12), within the same strong disequilibrium block. Despite this striking proximity, it has never been studied by whole-genome analysis perhaps because it is currently absent from the last human genome reference assembly. Notwithstanding the impact that the *RNU2* locus might have on *BRCA1*, the nearly total absence of recombination between these two loci can be used to follow *RNU2* alleles through many generations in *BRCA1* mutation carriers, and thus to estimate the timescale of evolutionary events.

We decided to explore more extensively the polymorphic state of macrosatellites by using, for the first time, the depth of coverage (DOC) of the *RNU2* repeat unit. To do so, we used data from the 1000 Genomes Project. We validated this approach by obtaining a very good correlation between the numbers of *RNU2* repeats estimated by DOC and by fiber–FISH in eight individuals. This method, that we recently developed in order to visualize the *RNU2* locus, is presently the most precise to directly count the allelic number of repeats (12). The wide variability of copy number we observed for 1106 individuals confirmed the extremely high level of polymorphism of this locus. We found a statistically different mean copy number in various recent modern human populations, favoring a scenario of fast evolution of this locus. We then studied a large number of meioses to determine more accurately the mutation rate of this macrosatellite by *BRCA1* mutation tracing rather than by investigating numerous trios. Contrasting with previous reports on macrosatellite extreme meiotic instability, we found that *RNU2* mutation rate in human modern population (5 × 10^−3^ per generation) is within the same range to that of microsatellites (estimated between 10^−5^ and 10^−2^ per locus per generation (15)).

## MATERIALS AND METHODS

### Cell lines

Human lymphoblastoid cell lines (LCLs) from eight CEPH individuals sequenced in the 1000 Genomes Project, chosen on the basis of their *RNU2* copy number, were purchased from the Coriell Institute for Medical Research (Camden, NJ, USA). Their reference number (and estimated copy number) are the following: NA12272 [11], NA12275 [12], NA07048 [20], NA12006 [32], NA11840 [40], NA06989 [52], NA07051 [71] and NA12718 [94]. The other studied LCLs were from subjects that belonged to *BRCA1* families and either carried the *BRCA1* mutation present in the family or were non-carriers. All LCLs were maintained in RPMI 1640 medium (Life Technologies, Saint Aubin, France) supplemented with 10% fetal calf serum (VWR, Fontenay sous Bois, France) and 1% penicillin–streptomycin (Life Technologies).

### Plug preparation, molecular combing of DNA and FISH

The procedure has been described in detail elsewhere (12). Briefly, Epstein Barr Virus (EBV)-immortalized lymphoblastoid cells were embedded in agarose blocks, DNA was purified, recovered from agarose and stretched on a silanized coverslip at constant speed (constant DNA stretching factor of 2 kb/μm). The quality of combing (linearity and density of DNA molecules) was estimated before performing hybridization with a bar code consisting of probes hybridizing to regions flanking the *RNU2* array, extending on the centromeric side up to *NBR1*, as well as probes hybridizing to parts of the *RNU2* repeat unit. Following the probe detection step, image acquisition was performed with a customized automated fluorescence microscope. Allelic number of copies was determined by measuring fiber length and by counting the number of signals corresponding to a repeat unit only on fibers for which intact flanking probes could be observed. Occasionally, a gap was observed in a random way within the *RNU2* array, most probably due to the absence of hybridization of one *RNU2* probe.

### *BRCA1* mutation age estimation

In order to estimate the age of the mutation (or more precisely, the number of generations since the most recent common ancestor, MRCA) of the carriers of the two mutations analysed in this study, we used the method that was first used to estimate the age of several *BRCA1* mutations (16). It was then extended and applied to *BRCA2* mutations (17) and used in several other similar studies, most recently in an analysis of the c.5266dupC *BRCA1* mutation (18). This method uses maximum likelihood and allows for both recombination and mutational events at the marker loci as means of altering a presumed ancestral haplotype. Phased haplotypes were used if these could be inferred from available family data; otherwise, all possible haplotypes were constructed from multi-locus genotype data and weighted according to their probability. For each value of G (the number of Generations since the MRCA), the relative likelihood that each haplotype is descended from the ancestral haplotype via mutation and recombination is calculated compared to the likelihood that it is a totally independent haplotype (i.e. an independent recurrent mutation on a different haplotype background). The value of G which maximizes this likelihood is obtained through iterative search. The 95% support intervals were constructed by identifying those points GL and GU where the likelihood differed from the maximum by 0.86 (corresponding to a chi-squared likelihood ratio statistic of 3.84, e.g. *p* = 0.05).

From the set of 323 SNPS in the *BRCA1* +/- 2MB region we selected 17 SNPs on the basis of linkage disequilibrium (LD) patterns and allele frequency to cover the region. These 17 SNPs spanned a region of 3.2 Mb. To obtain genetic positions of each marker analysed, we estimated the genetic position from the proportion of physical distance between the known markers and then translated this to the genetic scale, assuming 1 cM = 1 Mb. As our method uses marker allele frequencies in the calculation of the likelihood, we estimated these frequencies from a large sample of >10,000 *BRCA2* carriers genotyped for these SNPs as part of the iCOGS/CIMBA data.

### Determination of the number of *RNU2* repeat unit by using the Depth of Coverage value

DOC were calculated using mpileup (SAMtools) at position hs37d5:7,361,154-7,366,066 for the *RNU2* CNV repeat unit (avoiding interspersed sequences identified by RepeatMasker) and 17:41,400,862-41,401,838 for the right junction. The whole genome coverage has been estimated using the number of mapped bases according to data provided for each individual by the 1000 Genomes Consortium. The *RNU2* copy number per genome has been estimated by dividing the *RNU2* CNV repeat unit DOC by the whole-genome DOC and by doubling this value, giving the DOC-estimated *RNU2* copy number (DCN).

### Estimation of the *RNU2* mutation rate by maximum likelihood

As no information on the relationship between families was available, we assumed that those carrying the same *BRCA1* mutation radiated simultaneously from a single ancestor following a star phylogeny (Supplementary Figure S4).

The number of mutations along a lineage with *t* generations is denoted *X_t_* and follows a Poisson distribution with parameter *μt*, where *μ* is the mutation rate per generation. Because only a few generations were observed with low mutation rates, we chose to neglect reversions. We constructed the likelihood function *L(μ)* of the data as shown in formula [1], as we can count 639 generations without any mutation and independently 61, 73, 72 and 72 generations with at least one mutation occurring (Supplementary Figure S4).

[1] \begin{eqnarray*} L(\mu ) &=& p({\rm data}|\mu ) = p(X_{639} = 0) \times p(X_{61} \ge 1)\nonumber \\ && \times p(X_{73} \ge 1) \times p(X_{72} \ge 1) \times p(X_{72} \ge 1) \end{eqnarray*}

Because *p(X_t_ ≥ 1) = 1 - p(X_t_ = 0)*, the log-likelihood function *log(L(μ))* is:

[2] \begin{eqnarray*} \log \left( {L(\mu )} \right) &=& - 639\mu + \log (1 - e^{ - 61\mu } ) + \log (1 - e^{ - 73\mu } )\nonumber \\ &&+ \log (1 - e^{ - 72\mu } ) + \log (1 - e^{ - 72\mu } ) \end{eqnarray*}

We maximized this log-likelihood using the optimize function implemented in the R software with default parameters (19). We calculated the 95% confidence interval by inverting the acceptance region of the Wald test as described, e.g. in (20). This method relies on the asymptotic normality of the estimator. Visual inspection of the likelihood function suggested that the original parameterization did not lend itself well to this assumption. We therefore reparameterized our model using the log function to construct the interval and converted the interval back using the exponential function.

### Estimation of *RNU2* scaled mutation parameter *(θ = 4N_e_μ)*

We used two estimators $\hat \theta _{\bar x}$ and $\hat \theta _{NA}$ of the scaled mutation parameter *θ = 4N_e_μ*, where *N_e_* is the effective population size and *μ* is the mutation rate per generation developed previously for microsatellite loci (21). These estimators rely on a stepwise mutation model and were shown to accurately estimate *θ* from the total number of alleles *n_A_* or the mean allele frequency $\bar x$ in a sample. We used the allele distribution data described in two independent studies (11,12), leading to a total sample size of *n* = 504 haploid genotypes. These two studies identified *n_A_* = 53 different alleles with a mean allele frequency $\bar x = 0.0189$. Estimator $\hat \theta _{\bar x}$ is directly related to:

\begin{equation*} \hat \theta _{\bar x} = \frac{1}{{8\bar x^2 }} - \frac{1}{2}. \end{equation*}

$\hat \theta _{NA}$ is obtained from *n_A_* by solving for *θ* in the equation [7] of Haasl and Payseur (21):

\begin{equation*} n_A = (c_0 + c_1 ln(\theta ) + c_2 ln(\theta )^2 )\sqrt {1 + 2\theta } , \end{equation*}

where *c*_0_ to *c*_2_ are coefficients estimated through simulations that depend on sample size. We used the coefficient values provided for a sample size of *n* = 500: *c*_0_ = 2.0357, *c*_1_ = -0.07910 and *c*_2_ = -0.00007 (21). The equation was solved using the uniroot function of the R software (19).

## RESULTS

### Large diversity of *RNU2* copy number in 1000 Genomes Data

**Figure 1.**
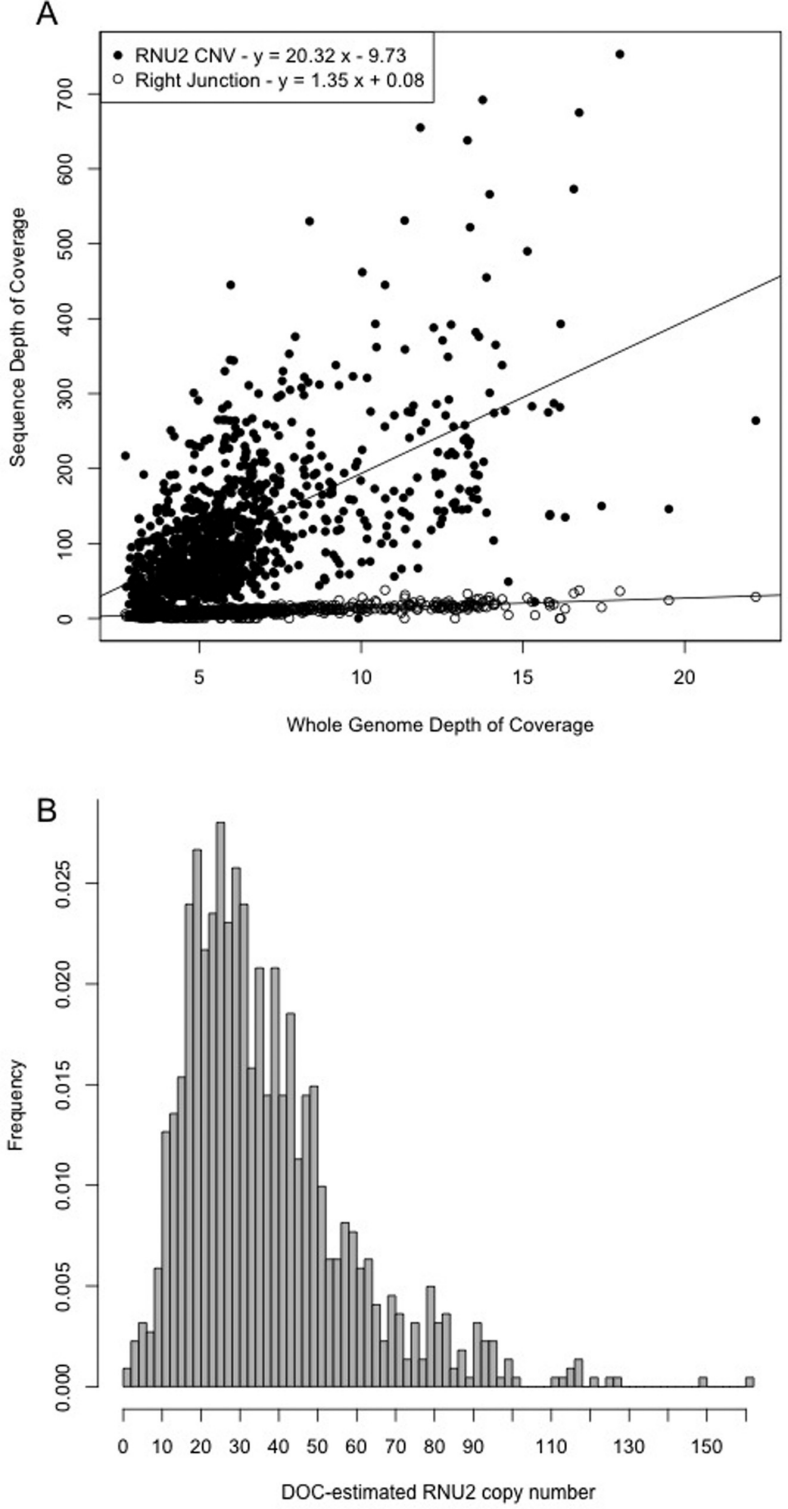
Estimation of *RNU2* copy numbers in 1106 individuals sequenced in the 1000 Genomes Project using the depth of coverage (DOC) value. (**A**) Sequence DOC for the *RNU2* repeat unit or the *RNU2* flanking region *versus* sequence DOC for the whole genome. (**B**) Distribution of DOC-estimated *RNU2* copy numbers (DCN).

**Figure 2.**
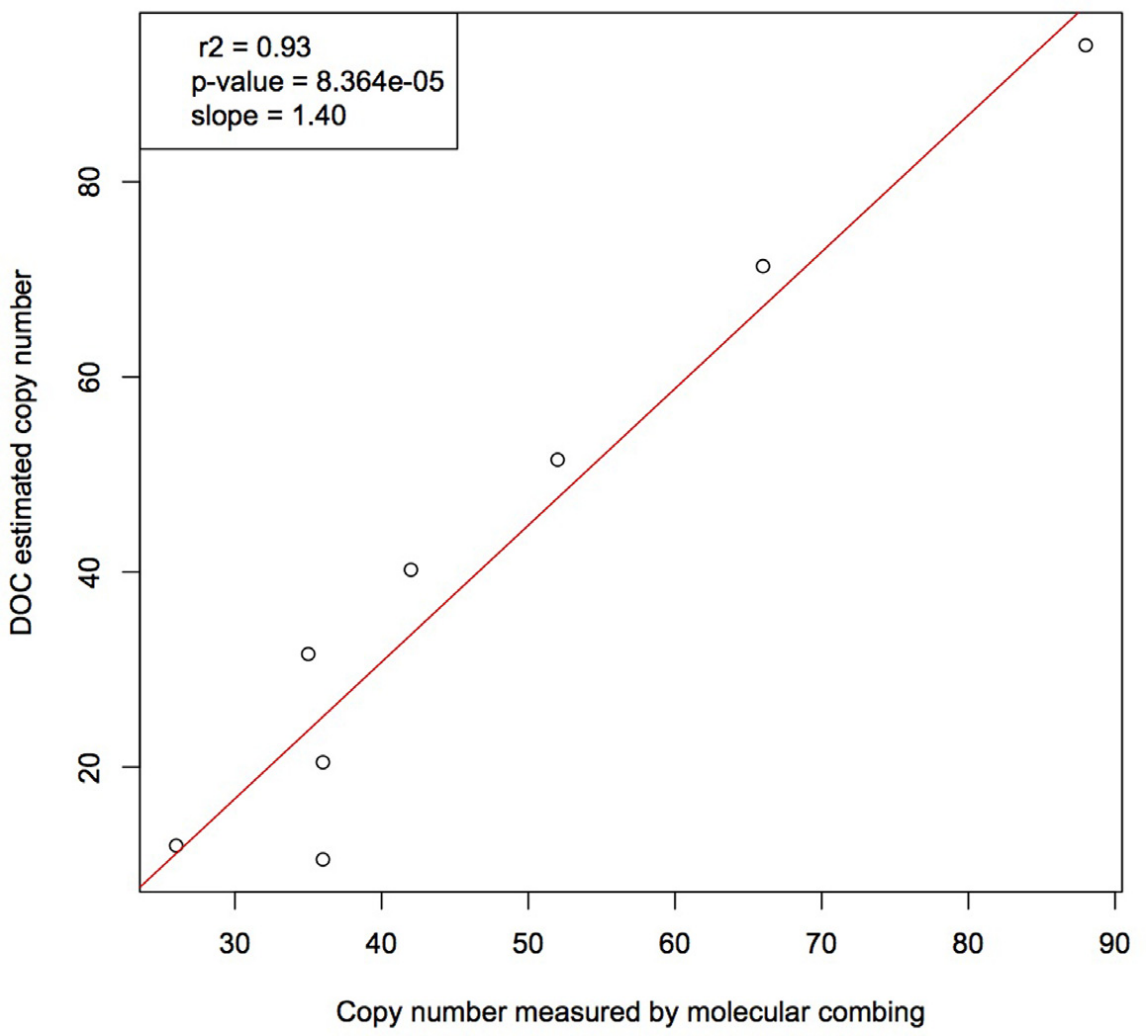
Correlation between DOC-estimated *RNU2* copy number (DCN) and *RNU2* copy number measured by molecular combing for eight individuals from the 1000 Genomes Project.

To estimate the degree of variability of the *RNU2* repeat unit copy number in a large set of individuals, we analysed the sequence data generated by the 1000 Genomes Project (1000 Genomes Phase 2). We mapped sequence reads obtained in a set of 1106 individuals using not only the reference genome assembly (Build 37/hg19) but also all known but unlocalized human genomic contigs (reference sequence set hs37d5) (22) that included at least one copy of the *RNU2* repeat unit. Sequence DOC of the *RNU2* repeat unit is systematically higher than that of whole genome DOC; by contrast, DOC of the macrosatellite right junction, which is not expected to be repeated, is not different than that of the whole genome (Figure 1A). By dividing the *RNU2* DOC by the whole genome DOC, we estimated the (diploid) *RNU2* copy number (DOC-estimated copy number, DCN). This DCN is on average 40.6, and is highly variable among individuals [2.5-160] (Figure 1B), which confirms that this locus is highly repetitive and polymorphic. To validate this macrosatellite genotyping approach, we first showed that the whole genome DOC has a negligible effect on the DOC-estimated copy number (*r*^2^ = 5 × 10^−3^, *p*-value = 0.0126), suggesting that the *RNU2* macrosatellite polymorphic status hence estimated is only weakly affected by sequence coverage. It is known that the sequencing coverage is not uniform across the genome and that it notably decreases in regions of extreme GC-content (23,24). Given that the *RNU2* repeat unit is GC-rich (GC content: ∼65%), the number of copy of this unit could be underestimated by the DOC method. To test this, we used a fiber–FISH approach that allows the number of *RNU2* repeat units to be precisely counted (12) in eight individuals with various *RNU2* DCN (10–94). We found a strong agreement between these two techniques (Figure 2, *r*^2^ = 0.93, *p*-value < 0.0001), suggesting that the *RNU2* DCN can be used as a surrogate for the true underlying *RNU2* copy number although we observed a slight underestimation of small arrays and overestimation of large arrays by DOC.

**Figure 3.**
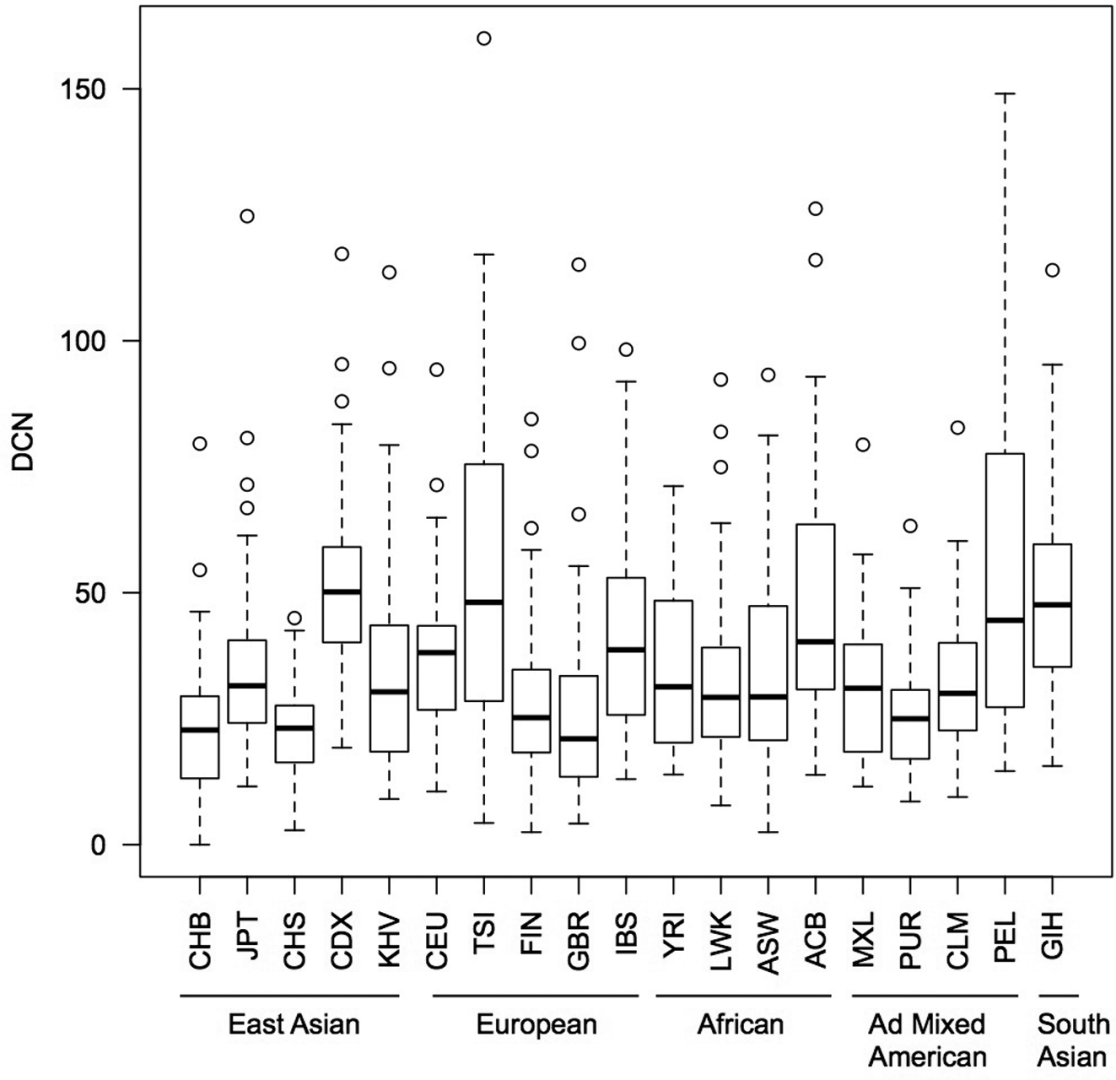
Mean *RNU2* copy numbers estimated by DOC in the different populations of the 1000 Genomes Project data. Black bars: median, Whiskers: interquartile. CHB: Han Chinese in Bejing China (N = 25); JPT: Japanese in Tokyo Japan (N = 65); CHS: Han Chinese South (N = 69); CDX: Chinese Dai in Xishuangbann China (N = 82); KHV: Kinh in Ho Chi Minh City Vietnam (N = 78); CEU: Northern Europeans from Utah (N = 42); TSI: Toscani in Italy (N = 11); FIN: Finnish in Finland (N = 65); GBR: British from England and Scotland (N = 59); IBS: Iberian populations in Spain (N = 76); YRI: Yoruba in Ibadan Nigeria (N = 46); LWK: Luhya in Webuye Kenya (N = 80); ASW: African Ancestry in Southwest US (N = 50); ACB: African Caribbean in Barbados (*N* = 55);MXL: Mexican Ancestry in Los Angeles California (*N* = 58); PUR: Puerto Rican in Puerto Rico (N = 63); CLM: Colombian in Medellin Colombia (N = 55); PEL: Peruvian in Lima Peru (*N* = 50);GIH: Gujarati Indian in Houston (*N* = 75). As for the 1000 Genomes Project, these populations have been divided into five super populations: East Asian (ASN), European (EUR), African (AFR), Ad Mixed American (AMR) and South Asian (SAN).

As the goal of the 1000 Genomes project is to to generate a comprehensive resource on human genetic variation in multiple human populations, we further explored variations in *RNU2* DOC in each of the five major population groups sequenced. Statistically significant differences in mean DOC-estimated *RNU2* copy numbers were seen between populations (Figure 3, Krustal–Wallis test *p*-value < 0.0001). Moreover, we observed a high diversity of distribution among super populations, such as, for example in Europe between the Tuscans from Italy (TSI) and the British from England and Scotland (GBR). Strikingly, the populations that seem to be among the lowest in copy numbers are two Chinese populations (CHB and CHS), while the highest values come from another Chinese population (CDX). We also observed a high heterogeneity between individuals within populations, especially for the Peruvians from Lima (PEL) and the Tuscans from Italia (TSI). Such diversity reflects the rapidity with which these sequences evolve.

### Transmission study of *RNU2* arrays in *BRCA1* families

As the mutation rate of macrosatellites is poorly documented, we next undertook to study the stability of the *RNU2* locus. This could be achieved by analysing many parent-to-offspring transmissions, a time-consuming task in the case of macrosatellites given that all the techniques currently available to genotype this category of polymorphisms (PFGE, FIGE or FISH on combed DNA) are low-throughput. Alternatively, one could follow alleles through a large timescale by genotyping distantly related individuals, the difficulty in this approach being the identification of such individuals. Here, we took advantage of the fact that the *RNU2* array and the *BRCA1* gene are located in the same haplotype block showing nearly complete LD (25), and of the identification of many *BRCA1* founder mutations indicating unsuspected familial relationships between apparently unrelated individuals. Indeed, the *BRCA1* LD block, first described in 1999 (25), has been delineated by the international HapMap project (August 2010 release) in Caucasians as a ∼290 kb-long interval comprising 23 kb centromeric and 185 kb telomeric to the *BRCA1* gene based on Build 37 assembly and dbSNP b126, thus comprising the *RNU2* locus. Consequently, recombinations between *BRCA1* and *RNU2* are expected to be extremely rare, making it possible to use *BRCA1* mutations to trace *RNU2* alleles.

**Figure 4.**
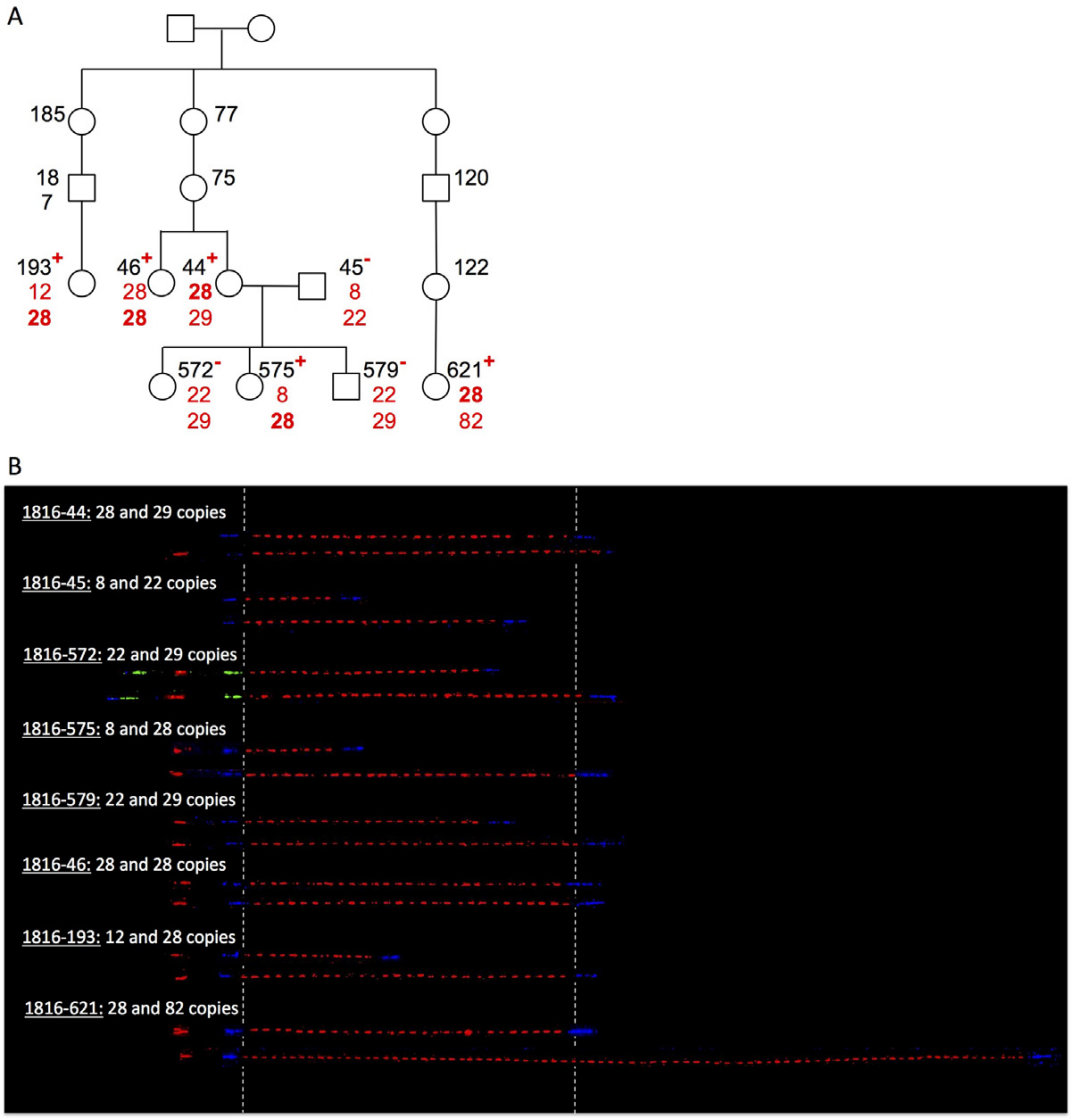
Inheritance of the *RNU2* array co-segregating with the c.4987-578_5074+342del1008 *BRCA1* mutation in a large *BRCA1* family. (**A**) Pedigree of Family 1816. The number of repeat units in *BRCA1* mutation carriers (^+^) or in individuals not carrying the *BRCA1* mutation (^−^) was determined by molecular combing and is shown in red, while the identification number of each individual is shown in black. The number of repeats shared by all the mutation carriers is bolded [28]. Only the family members that have been analysed in the present study are indicated for clarity. (**B**) Visualization by molecular combing and fiber–FISH of the 17q21 region around the *RNU2* macrosatellite. Probes hybridizing regions flanking the *RNU2* macrosatellite were labeled in green and/or blue, while a probe hybridizing a region within the *RNU2* array repeat unit was labeled in red. For each analysed individual, two fibers that display the bar code for the *RNU2* macrosatellite and flanking regions are shown, corresponding to the two alleles.

We first examined the stability of the *RNU2* array on a short timescale by measuring the number of repeat units segregating with a *BRCA1* mutation (c.4987-578_5074+342del1008, a 1-kb deletion comprising exon 17) in one of the largest *BRCA1* families reported in the literature, Family 1816. The pedigree records of this family extend over 6 generations, and include 56 *BRCA1* mutation carriers and 100 non-carriers. We chose to investigate *RNU2* arrays in eight family members carefully selected on the basis of them being either as closely or as distantly related as possible. As shown in Figure 4, allele segregation is strictly Mendelian in the nuclear family composed of two parents and three children (individuals 44, 45, 572, 575 and 579). The five mutation carriers analysed, separated by 1, 2, 5, 6 or 7 degrees, all share an allele carrying 28 repeat units, which indicates that the *RNU2* array displays meiotic stability on a short timescale and over a relatively small number of generations (at most 12 generations), at least for the array containing 28 repeat units.

We next measured the number of repeat units in the *RNU2* arrays segregating in individuals from different families but carrying the same *BRCA1* founder mutation. Two Ashkenazi Jewish founder mutations were studied: c.5266dupC (also known as 5382insC) and c.68_69delAG (also known as 185delAG), which have been estimated to have arisen, respectively, about 72 [49–107] and 61 [47-77] generations ago (18,26). We also analysed two other founder mutations for which no age estimation was available in the literature: c.4186-1787_4357+4122dup (also known as ins6kbEx13), identified in families originating from many different countries (27–31), and c.213-11T>G (also known as 332-11T>G). We therefore first undertook to estimate the number of generations since the MRCA in 34 carriers of c.4186-1787_4357+4122dup and 86 carriers of c.213-11T>G by using carrier genotypes for SNPs located in the *BRCA1* region, available thanks to the iCOGS study (32). The maximum-likelihood estimate of the time to the MRCA for the c.4186-1787_4357+4122dup haplotypes and c.213-11T>G haplotypes were 73 [52-100] generations and 87 [67-111] generations respectively.

**Figure 5.**
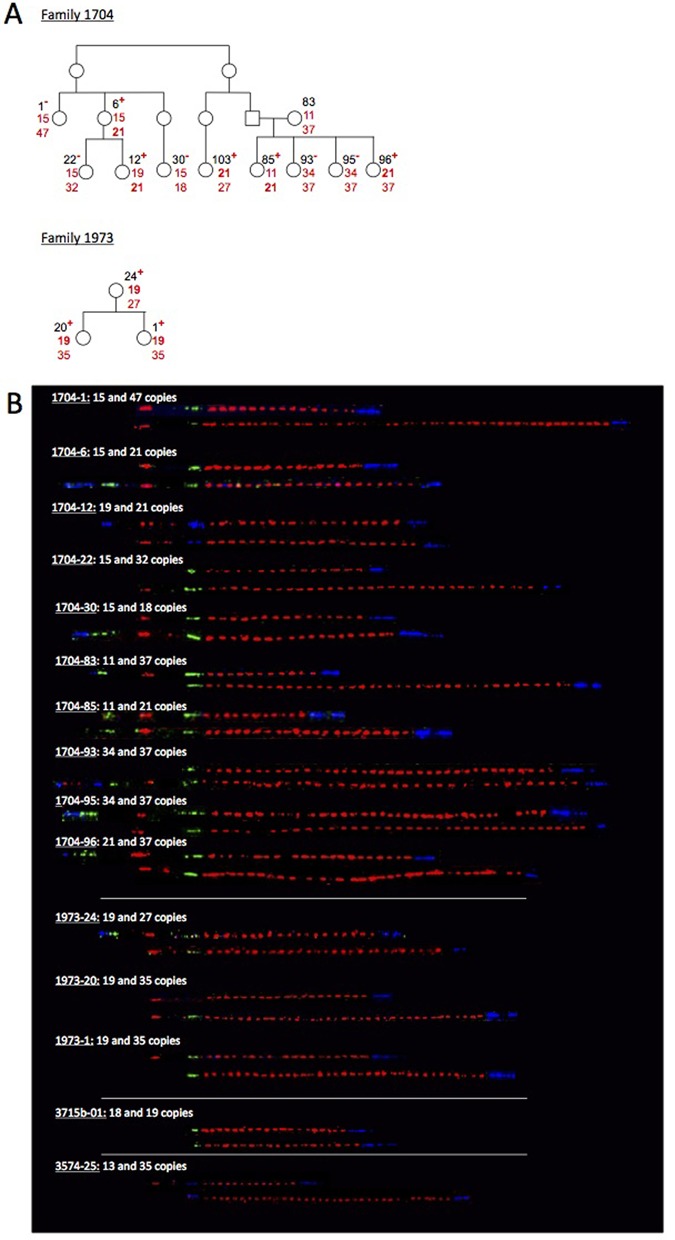
Inheritance of the *RNU2* array co-segregating with the c.5266dupC *BRCA1* mutation in the 1704, 1973, 3715b and 3574 families. (**A**) Pedigrees of the families for which more than one individual have been genotyped (F1704 and F1973). (**B**) Visualization by molecular combing and fiber–FISH of the 17q21 region around the *RNU2* macrosatellite. The rest of the legend is as in Figure 4.

The number of *RNU2* repeat units segregating with the *BRCA1* c.213-11T>G mutation, 22, is the same in all the carriers tested in two independent families (4 carriers in F2749 separated by 4 degrees at most; 2 carriers in F3103 separated by 5 degrees; Supplementary Figure S1). Conversely, different numbers of *RNU2* repeat units were shown to segregate with the three other *BRCA1* mutations. For some families, only one individual was available and could be genotyped. In these cases, the *RNU2* allele segregating with the *BRCA1* mutation was inferred, if possible. Concerning c.5266dupC for which carriers belonging to four independent families were tested, three different alleles were identified: 21 (in F1704), 19 (in F1973 and F3715b) and 35 or 13 (in F3574) *RNU2* repeat units (Figure 5). For c.68_69delAG, four independent families were studied and two different alleles were identified: 37 (in F2541, F3079 and F3261) and 47 (in F2979) repeat units (Supplementary Figure S2). For c.4186-1787_4357+4122dup, two different alleles carrying 29 and 14 repeats were identified in two independent families, F3173 and F3653 (Supplementary Figure S3). The results are summarized in Table 1.

**Table 1.** Overview of the results of copy numbers of the *RNU2* macrosatellite associated with various *BRCA1* mutations

*BRCA1* mutation	c.68_69 delAG	c.213-11T>G	c.4186-1787_4357 +4122dup	c.4987-578_5074 +342del1008	c.5266dupC
Age estimate (generations) [95% CI]	61 [47-77]^a^	87 [67-111]^b^	73 [52-100]^b^	-	72 [49–107]^c^
Number of independent families in CIMBA database	2097	56	45	1	2703
Number of countries in which the mutation has been identified in CIMBA database	25	6	6	1	24
Number of families analysed in the present study	4	2	2	1	4
Number of *RNU2* repeats associated with *BRCA1* mutation	37;47	22	14;29	28	13 or 35;19;21

^a^Estimated in (21).

^b^Estimated in this study.

^c^Estimated in (20).

To verify that recombinations between *BRCA1* and *RNU2* are indeed extremely rare and do not explain the few occurrences of variation in the number of *RNU2* repeats linked to some *BRCA1* mutations, we analysed SNPs genotypes for some of these individuals across the *BRCA1* LD block (Supplementary Table S1), available thanks to the iCOGS study (32). Complete LD was systematically observed between flanking polymorphic markers, thus showing that crossover between nonsister chromatids is extremely rare.

### Maximum-likelihood estimation of the mutation rate

To evaluate the mutation rate of the *RNU2* locus, we used a simple Poisson model of the *RNU2* mutation process, with a single mutation rate parameter *μ*, common to every allele. We used maximum likelihood to estimate this rate. To do so we needed to calculate the number of meioses where *RNU2* alleles were transmitted with unchanged copy numbers and the number of meioses where *RNU2* alleles were transmitted with different copy numbers. With this aim, we used estimates of the maximum number of generations that separate *BRCA1* carriers from their common ancestor, assuming that families are related by a star phylogeny (Supplementary Figure S4). Doing so, we expect to slightly underestimate the mutation rate as those individuals are likely to share a more recent common ancestor than assumed. Second, we determined the minimum number of *RNU2* mutations associated with each *BRCA1* founder mutation. As shown in Figures 4 and 5 and Supplementary Figures S1–S4, we observed at least 639 meioses without mutations (17 for F1816, 6 for F2749, 6 for F3103, approximately 174 generations between F2749 and F3103, 5 for F3173, 2 for F3653, 5 for F2979, 4 for F1973, and 20 for F1704, 183 between F3079/F2541/F3261 and their MRCA, approximately 174 generations between F2749/F3103 and their MRCA, 73 between F3173 or F3653 and their MRCA, 144 between F1973/F3715 and their MRCA). Conversely, we identified at least one mutational event between F3173 or F3653 and their MRCA (73 generations), one between F2979 and c.68_69delAG carriers’ MRCA (61 generations), one between F3514 and c.5266dupC carriers’ MRCA (72 generations) and one between F1704 and c.5266dupC carriers’ MRCA (72 generations). Using those data, we estimated the *RNU2* mutation rate to be 5 × 10^−3^ events per generation (95% confidence interval: [1.7 × 10^−3^ – 1.6 × 10^−2^]). We calculated again the *RNU2* mutation rate by taking into account the confidence intervals of the estimation of the mutation age and found it to be between 1.3 × 10^−3^ and 2.2 × 10^−2^.

We used a population genetics based approach to compare our estimates with independent estimates of the mutation parameter. We estimated the scaled mutation parameter *θ* = *4N_e_μ* at the *RNU2* locus in human populations from the total number of alleles *n_A_* and the average frequency of alleles $\bar x$, and compared it to the value of our mutation rate estimate. Assuming roughly *N_e_* = 10,000 for humans (33) leads to an estimate of $4N_e \hat \mu = 200$. Because *N_e_* in humans is hard to measure, *θ* could however be slightly higher (34). Using allelic distribution data taken from two independent studies (11,12) (total sample size *n* = 504), we obtained the following values: $\hat \theta _{\bar x}$ = 350.6 and $\hat \theta _{NA}$ = 602.2, which are slightly above but within the same range as our $4N_e \hat \mu$ estimate, 200. It is to be noted that neither of these estimates is higher than the value derived using the upper confidence limit of the mutation rate. These estimators are based on a stepwise model of satellite mutation with each step leading to an expansion or a reduction of copy number by one unit. This model also assumes no directional bias *i.e.* increases in copy number are as frequent as decreases. As for microsatellite, it is likely that this model is quite far from what really happens in the mutational process of macrosatellites. Indeed, we suspect that *RNU2* mutations lead mostly to multistep expansions (or reductions) in copy number because, as far as we know, some intermediate copy numbers (*e.g.* from 63 to 82) have never been found in any individual analysed yet, in all *RNU2* studies (11,12). Furthermore, the four mutations in the *BRCA1* data each involved far more than one repeat unit (and, statistically, these are unlikely to be multiple mutations). This discrepancy between the model and the suspected mutation behavior at *RNU2* locus is likely to explain why $\hat \theta _{\bar x}$ and $\hat \theta _{NA}$ do not provide values closer to each other. However, authors predicted that $\hat \theta _{\bar x}$ and $\hat \theta _{NA}$ are quite robust to the violation of the one-step assumption (21) making them the best choices currently available to simply compare our estimate of *μ* to previous results.

## DISCUSSION

The study of macrosatellites is highly challenging. Not only it is very difficult to identify this type of multi-allelic CNV due to their absence from the genome reference assembly, but their genotyping is also problematic. Techniques that can be used presently are either time- and material-consuming and necessitate high skills (PFGE, FIGE or FISH on combed DNA), or are high-throughput but cannot resolve allelic copy number (qPCR). The determination of the spectrum and frequency of their allelic variations in a population is therefore difficult at the present time, especially as they are each likely to have hundreds of different alleles. For the time being, it seems that large-scale variability is approachable through global copy number determination only.

While high-resolution sequence data generated by next-generation sequencing has been widely used for CNV detection, it had never been used, to our knowledge, to gain more insight into the variability of macrosatellites. We have shown here for the first time that DOC gives an accurate estimation of macrosatellite copy number. Indeed, we found a good agreement of the figures obtained by this approach with the numbers of *RNU2* repeat units measured by fiber–FISH for eight individuals with various values of *RNU2* DOC. Having shown that global copy numbers of the *RNU2* repeat are highly variable in the 1106 individuals sequenced in the 1000 Genomes Project, we noticed statistically significant differences in mean *RNU2* copy numbers estimated by DOC between populations. We concluded from this observation that the *RNU2* macrosatellite evolves rapidly and decided to further investigate the mutation rate of this locus. The localization of the *RNU2* locus within the *BRCA1* LD block gave us the unique opportunity to follow *RNU2* arrays through numerous meioses spread over a large period of time, as very large *BRCA1* kindreds and several *BRCA1* founding mutations have been identified. We analysed the transmission of *RNU2* arrays associated with five different *BRCA1* mutations, one ‘private’ mutation identified in a single extended kindred and four founder mutations. Although their age estimate is more or less equivalent, their frequency is highly variable (Table 1). Indeed, c.68_69delAG and c.5266dupC, the two founder Ashkenazi Jewish mutations, were reported each in more than 2000 families while c.213-11T>G and c.4186-1787_4357+4122dup were reported in 56 and 45 families, respectively, in the Consortium of Investigators of Modifiers of *BRCA1/2* (CIMBA) database (L. McGuffog, personal communication), which contains the world's largest collection of *BRCA1/2* carriers originating from 41 countries on six continents. Neither in the 13 parent-to-child transmissions that we analysed in total nor in *BRCA1* c.213-11T>G or c.4987-578_5074+342del1008 mutation carriers did we identify any copy number alteration of the *RNU2* array associated with the *BRCA1* mutation. However, c.68_69delAG and c.4186-1787_4357+4122dup were associated with two different numbers of repeats and c.5266dupC, the most frequent mutation, with three.

Using this innovative approach, we estimated the mutation rate of this locus in human modern population to be about 5 × 10^−3^ per generation, a mutation rate close to that of microsatellites (10^−5^ to 10^−2^ per locus per generation (15)). Our estimation is in agreement with the only published data concerning the *RNU2* locus stability which, although limited to the Mendelian transmission of the number of repeat units in one two-generation and one three-generation families, showed a mutation rate of 8 × 10^−3^ per generation (35,36). It also fits with the allelic diversity reported at this locus by our team and another previous study (11,12). On the other hand, this *RNU2* mutation rate contrasts with both meiotic and mitotic instability of the few macrosatellite sequences studied to date. Indeed, generational transmission studies of a few macrosatellite sequences revealed a high frequency of meiotic instability. For the SST1 macrosatellite that displays 134 different alleles carrying 14 to 154 copies of a 2.4-kb repeat unit, changes in copy number during parent to offspring transmission was evidenced in one of three three-generation families (with either six or seven children in the last generation) (9). For the TAF11-Like macrosatellite (54 different alleles carrying 10-98 copies of a 3.4-kb repeat unit), one of two three-generation families (with either six or seven children in the last generation) showed copy number alteration in meiotic transmission. In the case of RS447 for which more than 50 distinct alleles carrying 8-113 copies of a 4.7 kb repeat unit have been described, one study of 60 parent-to-offspring transmission reported an extremely high frequency (∼8.3%) of meiotic instability (10) while another one carried on 60 trios found no intergenerational copy number alteration (11). In our study, macrosatellite stability was investigated using the molecular combing technology rather than PFGE as done previously, allowing determination of unit repeat numbers independently of restriction enzyme digestion that can be skewed by SNP through restriction enzyme site modification. However, we do not believe that this might explain why our results are different. Rather, we favor the hypothesis that the apparent higher stability of the *RNU2* macrosatellite might be due to its location within a region of low recombination, contrary to RS447, SST1 and TAF11-Like.

It is interesting to note that such a relatively stable macrosatellite sequence still has many different alleles and displays a high level of heterozygosity, suggesting that unequal crossing over is not the principal mechanism generating new macrosatellite alleles. In the case of the *RNU2* locus, the molecular mechanism through which repeat copy number is altered is most likely unequal sister chromatid exchange as we were able to confirm through a haplotype analysis that there is no exchange between flanking markers. It has long been known that interchromosomal genetic exchanges are rare at the *RNU2* locus and that reciprocal nonsister chromatid exchange apparently does not occur, thanks to investigations on the concerted evolution of the *RNU2* repeats (35). Besides, it must be said that the mechanisms that lead to changes in copy number in this specific class of structural variation have been poorly documented although progress has been made in the understanding of the mutability of microsatellites (37,38) and complex human genomic rearrangements (39). Hopefully, new technological and analytical developments will soon make it possible to study macrosatellites more easily. Indeed, detailed characterization of macrosatellites and elucidation of their mutational dynamics are an important step toward a better understanding of the genetic instability of the genome and the potential association of structural variations with complex diseases and evolution.

## SUPPLEMENTARY DATA

Supplementary Data are available at NAR Online.

SUPPLEMENTARY DATA
