# Supplementary material for: Estimation of the RNU2 macrosatellite mutation rate by BRCA1 mutation tracing
Source: Nucleic Acids Res. 2014 Jul 17;42(14):9121–30. doi: 10.1093/nar/gku639 (PMC4132748; doi:10.1093/nar/gku639)
Supplement: SUPPLEMENTARY DATA [file supp_42_14_9121__index.html]

Estimation of the RNU2 macrosatellite mutation rate by BRCA1 mutation tracing — SUPPLEMENTARY DATA 

# Estimation of the *RNU2* macrosatellite mutation rate by *BRCA1* mutation tracing

## SUPPLEMENTARY DATA

**Files in this Data Supplement:**

- SUPPLEMENTARY DATA
- SUPPLEMENTARY DATA
