# Supplementary material for: Estimation of the RNU2 macrosatellite mutation rate by BRCA1 mutation tracing
Source: Nucleic Acids Res. 2014 Jul 17;42(14):9121–30. doi: 10.1093/nar/gku639 (PMC4132748; doi:10.1093/nar/gku639)
Supplement: SUPPLEMENTARY DATA [file supp_gku639_nar-00908-d-2014-File007.pdf]

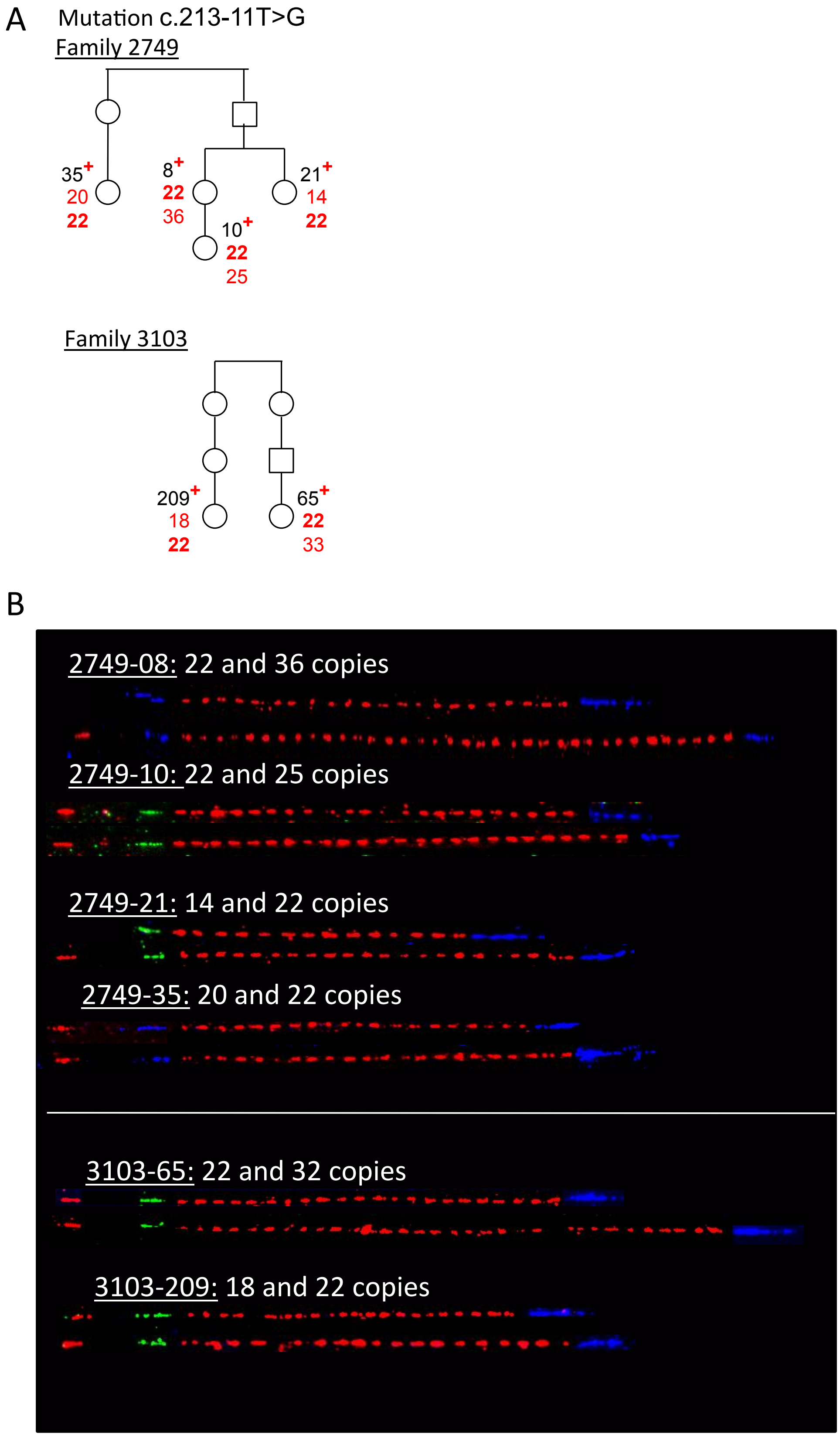

Supplementary Figure 1. Inheritance of the *RNU2* array co-segregating with the c. 213-11T>G *BRCA1* mutation in 2 *BRCA1* families. (A) Pedigrees of Family 2749 and Family 3103. The number of repeat units in *BRCA1* mutation carriers (+) was determined by molecular combing and is shown in red, while the identification number of each individual is shown in black. The number of repeats shared by all the mutation carriers is bolded (22). Only the family members that have been analyzed in the present study are indicated for clarity. (B) Visualization by molecular combing and fibre-FISH of the 17q21 region around the *RNU2* macrosatellite. Probes hybridizing regions flanking the *RNU2* macrosatellite were labeled in green and/or blue, while a probe hybridizing a region within the *RNU2* array repeat unit was labeled in red. For each analyzed individual, two fibers that display the bar code for the *RNU2* macrosatellite and flanking regions are shown, corresponding to the two alleles.

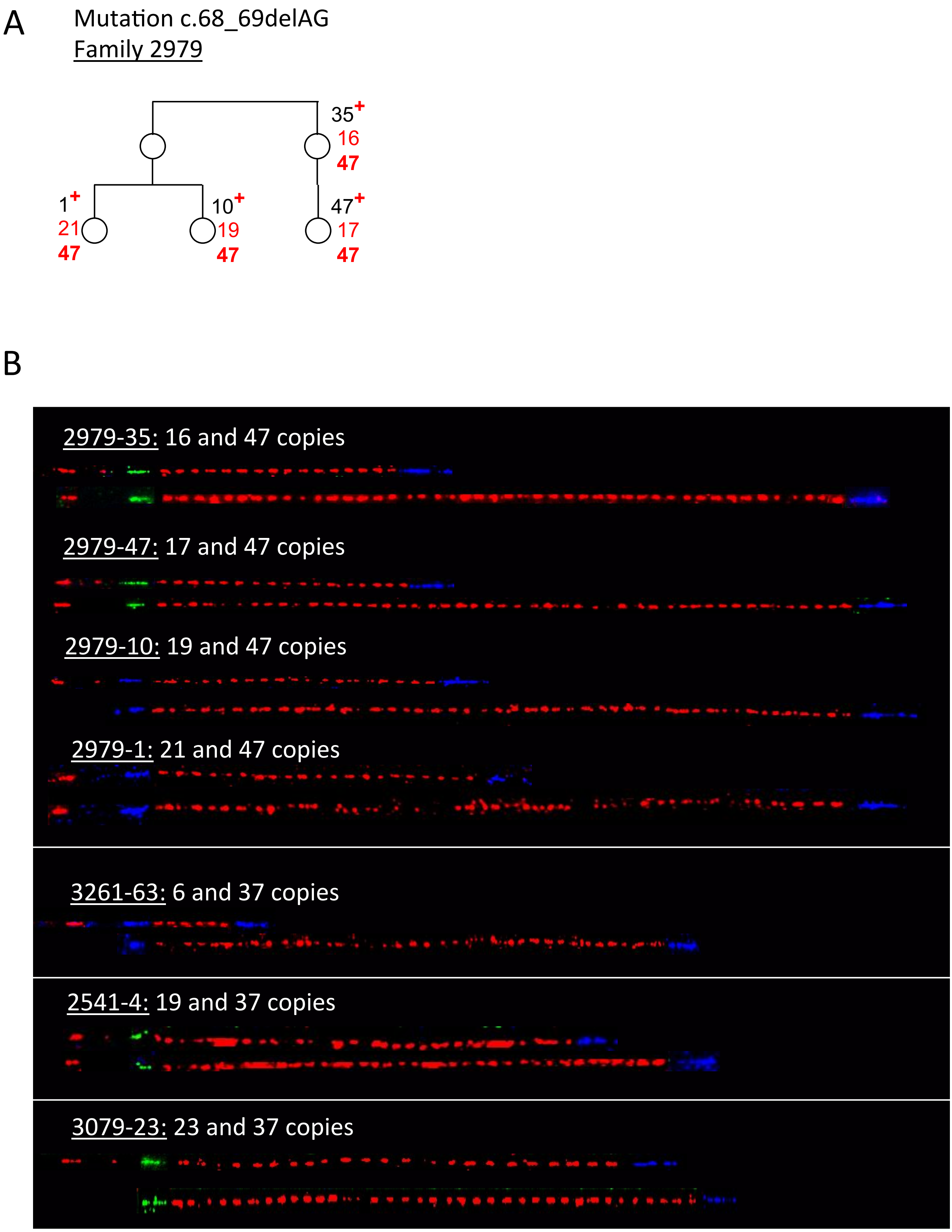

Supplementary Figure 2. Inheritance of the *RNU2* array co-segregating with the c. 68_69delAG *BRCA1* mutation in 4 *BRCA1* families. (A) Pedigree of Family 2979. (B) Visualization by molecular combing and fibre-FISH of the 17q21 region around the *RNU2* macrosatellite.

The rest of the legend is as in Figure Supp1.

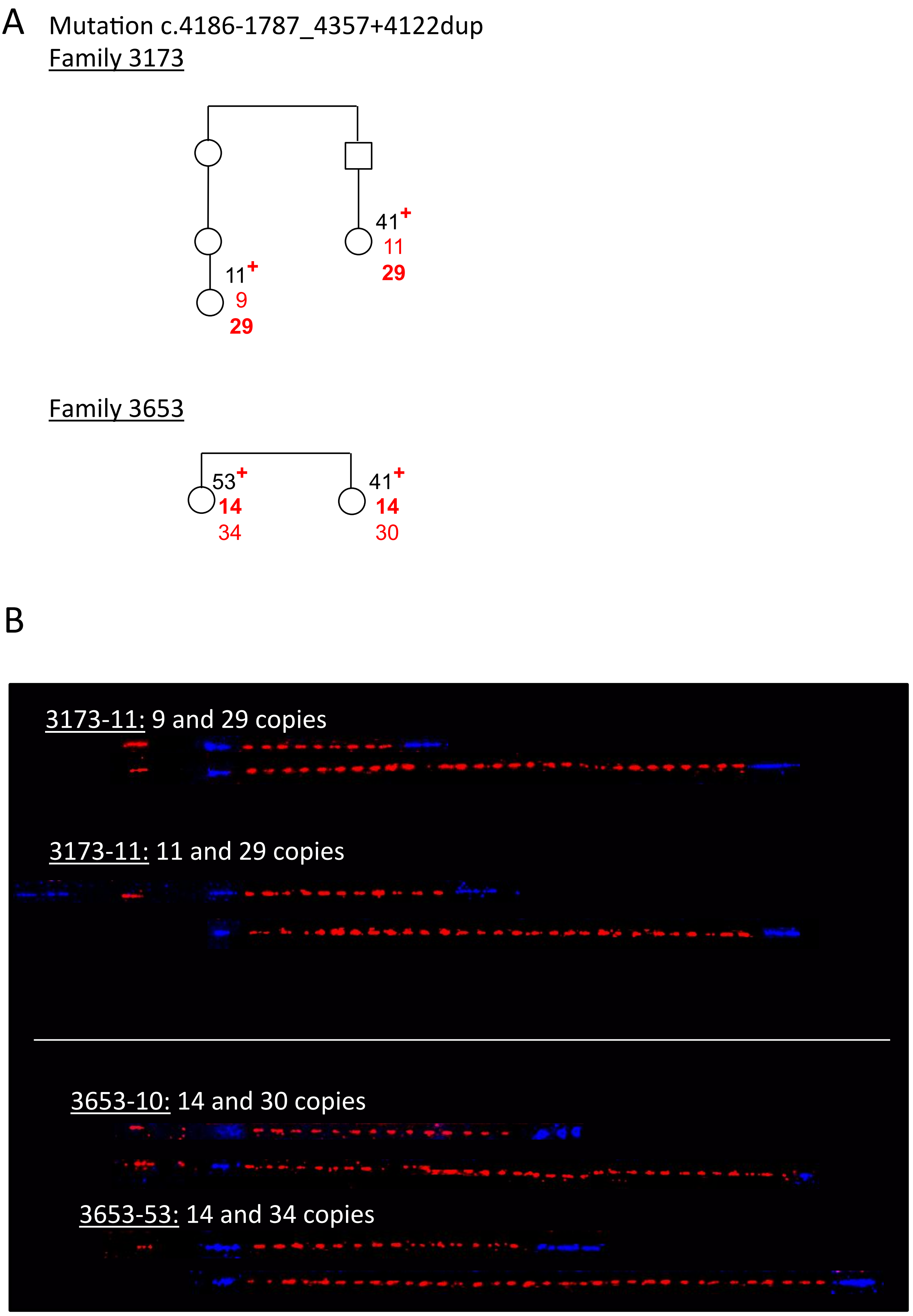

Supplementary Figure 3. Inheritance of the *RNU2* array co-segregating with the c. 4186-1787_4357+4122dup *BRCA1* mutation in 2 *BRCA1* families. (A) Pedigrees of Family 3173 and Family 3653. (B) Visualization by molecular combing and fibre-FISH of the 17q21 region around the *RNU2* macrosatellite.
The rest of the legend is as in Figure Supp1.

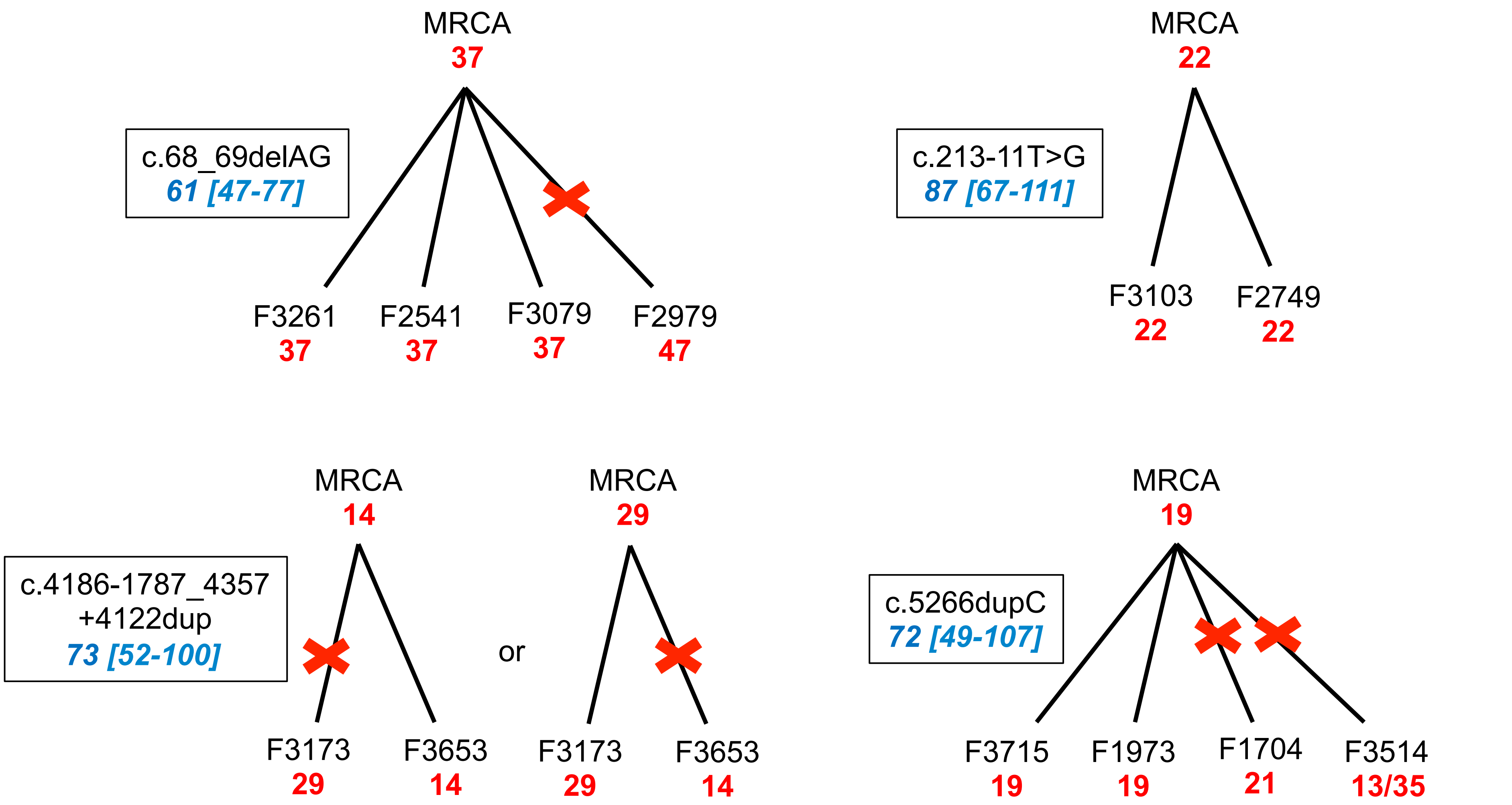

Supplementary Figure 4. Phylogeny of alleles of the *RNU2* macrosatellite associated with *BRCA1* founder mutations. In the absence of information, we assumed that families (F) carrying the same *BRCA1* mutation are connected by a star phylogeny . The maximum number of generations that separates *BRCA1* carriers from their most recent common ancestor (MRCA), i.e. the age of the mutation, is indicated in blue italic. The number of *RNU2* repeats associated with the *BRCA1* mutation for each family is indicated in red. Mutations of *RNU2* alleles are represented by red crosses.
